# Plateletcrit for predicting prognosis in patients with hepatic sinusoidal obstruction syndrome caused by pyrrolizidine alkaloid

**DOI:** 10.1002/jcla.24240

**Published:** 2022-01-16

**Authors:** Hongfei Tu, Miao Li, Zhiyuan Chen, Jiamin Zhao, Huali Wang, Jingjing Qian, Cheng Wei, Yang Yang, Yue Zhu, Lingyun Zuo

**Affiliations:** ^1^ Department of Gastroenterology The Second Hospital of Nanjing Nanjing University of Chinese Medicine Nanjing China; ^2^ Department of Laboratory Medicine Nanjing Drum Tower Hospital Nanjing University Medical School Nanjing China; ^3^ School of Medicine &Holistic Integrative Medicine Nanjing University of Chinese Medicine Nanjing China

**Keywords:** hepatic function evaluation, hepatic sinusoidal obstruction Syndrome, inflammation, plateletcrit

## Abstract

**Background:**

Platelet index was reported to be used as a potential prognostic marker in patients with liver fibrosis. We aimed to explore the association between plateletcrit (PCT) and severity of hepatic sinusoidal obstruction syndrome (HSOS).

**Methods:**

Seventy consecutive patients who diagnosed as HSOS by CT and medical history during January 2017‐November 2021 were included. All patients were divided into two groups which confirmed as favorable prognosis and poor prognosis on the basis of Child‐Turcotte‐Pugh score system. The clinical manifestation and laboratory parameters of two groups were retrospectively selected. PCT was evaluated within two groups, and the diagnostic accuracy was evaluated by the area under the receiver operating characteristic curve.

**Results:**

The significant difference between the two groups not only in diarrhea, abdominal pain, abdominal distention, urine volume, and skin ecchymosis (*p *< 0.005), but also in WBC count, NE count, PLT count, TBIL, and D‐Dimer (*p* < 0.005) were found. The PCT level was significantly higher in HSOS patients with poor prognosis (0.169Â ± 0.060) than favorable prognosis patients (0.110Â ± 0.047). The area under the receiver operating characteristic curve of RDW in predicting poor prognosis was 0.781, with 67.70% sensitivity and 79.5%specificity.

**Conclusions:**

The PCT level was correlated positively with the poor prognosis in HSOS patients. PCT can be a promising indicator for predicting prognosis in HSOS

## Graphical abstract

Regarding the poor prognosis group as the study groups and the good prognosis group as the control group, the ROC curve of PCT was constructed. The analysis of the ROC curve showed that the area under the ROC curve of PCT for poor prognosis of HSOS was 0.781 and the Youden index was 0.472. When the value of PCT was 0.135, it had the greatest diagnostic value for poor prognosis of HSOS patients. At this time, the sensitivity and specificity were 67.7% and 79.5%, respectively.

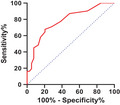

## 1 INTRODUCTION

In recent years, the epidemiology of liver diseases has changed with the development of diagnosis and treatment technology. Hepatic sinus obstruction syndrome (HSOS), also known as hepatic veno‐occlusive disease (HVOD), is an obliterative phlebitis of the terminal hepatic venules that has gradually attracted extensive attention. In developed countries, HSOS usually occurs not only after hematopoietic stem cell transplantation (HSCT) pretreatment,^1^ but also after chemotherapy for acute myeloid leukemia.^2^ In China, the main cause of HSOS is the oral administration of traditional Chinese medicine containing pyrrolizidine alkaloids, such as Herb of Chrysanthemum‐like Groundsel.^3^, ^4^, ^5^ Therefore, this type of HSOS is also called pyrrolizidine alkaloid associated hepatic sinusoidal obstruction syndrome (PA‐HSOS). The clinical manifestations include acute manifestations of rapid and massive abdominal swelling and pain related to hemorrhagic central lobular necrosis and jaundice, subacute recurrent ascites, hepatosplenomegaly associated with extensive fibrosis in the central area of the lobules, and a chronic variant of venocentric cirrhosis.^6^

The pathophysiological mechanism of hepatic veno‐occlusive disease or sinusoidal obstruction syndrome (VOD/SOS) is believed to be the initial toxic damage to the sinusoidal endothelium caused by a variety of factors, which trigger the activation and damage of endothelial cells, resulting in an increased permeability of the sinusoidal barrier.^6^, ^7^, ^8^ This primary endothelial injury allows red blood cells, leukocytes, and other cell debris to be deposited in the space of Disse, which may lead to a pathophysiological cascade, including loss of thrombo‐fibrinolytic balance, further endothelial detachment, downstream embolization, and occlusion of microcirculation.^6^, ^7^, ^8^, ^9^, ^10^ These events may lead to hepatorenal hypertension and cause multiple organ dysfunction (MOD).^6^, ^8^, ^9^

There is growing recognition of the critical role of platelets in inflammation and immune responses of various diseases, such as nasal polyps,^11^ type 2 diabetes mellitus,^12^ lumbar disc hernias,^13^ autoimmune liver diseases,^14^ and irritable bowel syndrome.^15^ Plateletcrit is also one of these platelet parameters and is associated with various conditions, including rheumatoid arthritis^16^ and papillary thyroid cancer.^17^ At present, it remains challenging to use blood indicators to predict the prognosis of HSOS, especially in an earlier stage. It is reported that platelet hematocrit (PCT) is a potential index for evaluating liver fibrosis and deterioration of liver function.^6^, ^7^ However, the association between PCT and HSOS remains largely unknown. Thus, this study aimed to investigate the relationship of PCT and the severity of HSOS disease to predict the prognosis of HSOS in the early stage.

## 2 PATIENTS AND METHODS

### 2.1 Patients

In this study, 200 patients with jaundice, hepatomegaly, and ascites admitted to The Second Hospital of Nanjing from July 2017 to September 2021 were selected as the research objects. The inclusion criteria are detailed in the Nanjing criteria in Table 1. The exclusion criteria are as follows: (a) Patients with other liver diseases, such as viral hepatitis, primary biliary cirrhosis, nonalcoholic steatohepatitis, autoimmune hepatitis, drug‐induced liver disease, metabolic liver disease, or alcoholic liver disease, (b) have hepatocellular carcinoma (HCC) or other tumors, (c) suffer from serious primary diseases of other systems, including heart, kidney, respiratory system, immune system, blood system, and mental sickness, (d) pregnancy, and (e) a history of hematopoietic stem cell (HSCT) transplantation.

**TABLE 1:** Requirements of modified Seattle criteria and Nanjing criteria

Standard	Diagnosis	Requirements			
		1	2	3	4
Modified Seattle criteria	HSCT‐HSOS	20 days post‐stem cell transplantation; at least meet 2 of the following 3 items	Hepatomegaly or right upper quadrant pain of liver origin	Jaundice (total serum bilirubin 2 mg/dl)	Unexplained weight gain (2% of baseline body weight) because of fluid accumulation
Nanjing criteria	PA‐HSOS	Taking herbs containing PA, at least meet 2 of the following 3 items, or pathological diagnosis and other cause induced liver injury	Hepatomegaly or right upper quadrant pain of liver origin, ascites	Increased serum total bilirubin or other abnormal liver function	Typically contrast‐enhanced CT or MRI manifestation

### 2.2 Diagnostic and subgroup criterion

Currently, the international clinical diagnostic criteria for HSCT‐HSOS mainly include the revised Seattle Criteria and Baltimore Criteria.^18^ However, the sensitivity and specificity of the above two criteria have not been fully verified in the diagnosis of HSOS caused by other reasons.^19^ Therefore, this study implemented the “Nanjing Standard”^20^ for the diagnosis of PA‐HSOS (Table 1). The hepatic histological changes in SOS include damage to the hepatocytes and sinusoidal endothelial cells in the central lobular zone (zone 3) of the liver acinus, leading to subendothelial edema in hepatic venules, fibrin deposition, fibrin microthrombosis, venular narrowing and sclerosis, and sinusoidal fibrosis, followed by hepatocyte necrosis.^6^, ^9^ Typical CT/MRI shows Patchy liver enhancement and heterogeneous hypoattenuation in contrast‐enhanced portal phase CT scans.^21^

### 2.3 Group criteria

The Child‐Turcotte‐Pugh (CTP) scoring system is widely used to predict the prognosis of patients with cirrhosis. It contains three objective symptoms (serum bilirubin, albumin concentration, and prothrombin time) and two subjective symptoms (ascites and encephalopathy) of liver failure. The CTP scores range from 5 to 15 points and are divided into three grades, including grade A (5–6 points), grade B (7–9 points), and grade C (10–15 points).^22^ According to the CTP score, all enrolled patients were divided into two groups, with good prognosis in grade A and B and poor prognosis in grade C.

### 2.4 Clinical and laboratory data

We retrospectively reviewed the demographic characteristics and laboratory results of the enrolled patients, including age, sex, platelet (PLT), PCT, white blood cell count, liver enzymes, total bilirubin, albumin, prothrombin time (PT), and D‐Dimer. The three objective symptoms and two subjective symptoms related to CTPS were abstracted and calculated at the end of hospitalization.

### 2.5 Statistical analysis

The continuous variables were expressed as mean ± standard deviation or median (iqr) and the categorical variables were displayed as counts and percentages. Kolmogorov‐Smirnov test was used to detect the normal distribution of the continuous variables. The independent group *t* test or Mann–Whitney U was used to compare continuous variables between groups. Chi‐square or Fisher's exact test was used to compare categorical variables. *p *< 0.05 was considered statistically significant. The receiver‐operating characteristic curve was used to evaluate the diagnostic performance of predictors of significant liver inflammation. The area under the receiver‐operating characteristic curve (AUROC) and the 95% confidence interval (CI) of AUROC were calculated. If AUROC > 0.7, it indicated that the index has certain diagnostic value and can be used for clinical reference. All data analyses were performed using SPSS version 22.0 software (SPSS Inc.).

## 3 RESULTS

### 3.1 Baseline characteristics of two groups

**FIGURE 1:**
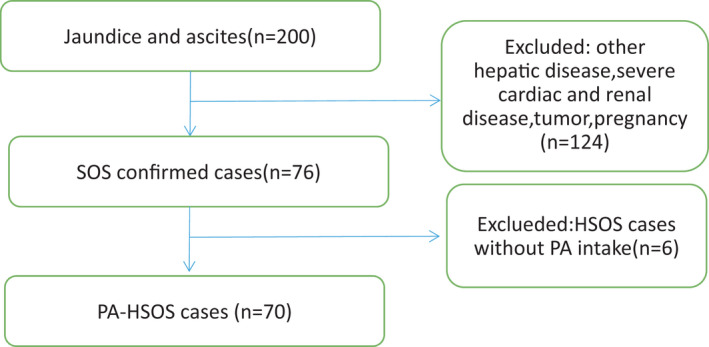
Process of case screening

In this study, 76 SOS patients were selected from 200 patients with liver injury. After the exclusion of six patients (5 patients were treated with HSCT and 1 patient had taken oxaliplatin), 70 patients were eventually diagnosed as PA‐HSOS (Figure 1).

Based on the PA‐HSOS diagnostic criteria and the Child‐Turcotte‐Pugh scoring system, the enrolled 70 patients were divided into two groups. The favorable prognosis group contained 17 cases of grade A (5–6 points) and 22 cases of grade B (7–9 points), and the poor prognosis group had 31 cases of grade C (Table 2). There were 33 men and 6 women in the favorable prognosis group with an average age of 61.77 ± 8.74 years, while the other group had 22 men and 9 women with an average age of 61.23 ± 10.42 years (Table 3).

**TABLE 2:** Child‐Turcotte‐Pugh scores of the two groups at the end of hospitalization

Groups	CTP scores	Score	HE	ASCITES	TBIL	ALB	PT
Favorable prognosis	Grade A (5–6 points) *n* = 17	1	17	10	8	6	12
		2	0	7	6	12	7
		3	0	0	3	1	0
	Grade B (7–9 points) *n* = 22	1	22	16	13	12	10
		2	0	5	7	9	2
		3	0	1	2	1	10
Poor prognosis	Grade C (≥10 points) *n* = 31	1	29	23	26	20	30
		2	2	8	5	11	1
		3	0	4	11	5	11

**TABLE 3:** Laboratory findings of the two groups at the beginning of hospitalization

Parameters	Groups		*p*‐Value
	Poor prognosis	Favorable prognosis	
SEX (M/F)	22/9	33/6	0.167
AGE (years old)	61.23 ± 10.42	61.77 ± 8.74	0.813
WBC (109/L)	8.21 ± 5.28	5.97 ± 2.19	0.033
NE (109/L)	5.84 ± 4.77	3.82 ± 1.73	0.031
PLT (1012/L)	368 ± 59.68	310 ± 47.42	0.026
ALT (U/L)	54.10 (24.10,146.30)	47.80 (26.90,126.20)	0.175
AST (U/L)	134.10 ± 153.30	148.87 ± 152.50	0.689
TBIL (umol/L)	56.10 (36.40,82.20)	35.80 (24.30,50.30)	0.012
PT (second)	17.82 ± 9.61	16.71 ± 7.70	0.595
PTA	57.60 ± 21.88	59.40 ± 19.65	0.718
HCT	41.97 ± 8.40	42.28 ± 6.36	0.862
ALB (g/L)	32.12 ± 5.54	33.76 ± 5.03	0.199
INR	1.80 ± 2.45	2.18 ± 2.99	0.634
D‐Dimer (mg/L)	1.78 ± 1.49	3.76 ± 2.35	0.002

### 3.2 Clinical manifestations and laboratory data

The clinical data of the enrolled patients at the initial stage of onset were collected and summarized as follows: hepatic hemangioma (3/70), type 2 diabetes (10/70), hypertension (8/70), rheumatoid arthritis (1/70), cholecystolithiasis (10/70), renal insufficiency (4/70), coronary atherosclerotic heart disease (2/70), gouty liver cyst (3/70), renal cyst (4/70), chronic cholecystitis (5/70), rectal polyps (1/70), and thyroid nodules (1/70). In addition to the common jaundice and ascites, other clinical manifestations are shown in Table 4.

**TABLE 4:** Clinical symptoms of the two groups at the beginning of hospitalization

Parameters	Groups		*p*‐Value
	Favorable prognosis (*n* = 39)	Poor prognosis (*n* = 31)	
Fatigue	32	29	0.153
Nausea	17	19	0.141
Diarrhea	10	15	0.049
Abdominal pain	22	25	0.032
Abdominal distension	26	31	0.000
Abdominal circumference (cm)	99.3 ± 10.24	110 + 22.34	0.011
Urine volume (ml)	1500 ± 240	1000 ± 330	0.000
Edema of lower limbs	32	31	0.013
Skin ecchymosis	22	29	0.001

### 3.3 Comparison of PCT levels in the two groups

As shown in Table 5, we compared the PCT levels of the two groups and analyzed the PCT with Wilson correlation index. The result showed that OR = 0.489.

**TABLE 5:** PCT level comparison of two groups

Groups	Case	PCT	*t*	*p*
Poor prognosis	31	0.169 ± 0.060	4.579	0.000
Favorable prognosis	39	0.110 ± 0.047		

### 3.4 PCT for predicting poor prognosis

**FIGURE 2:**
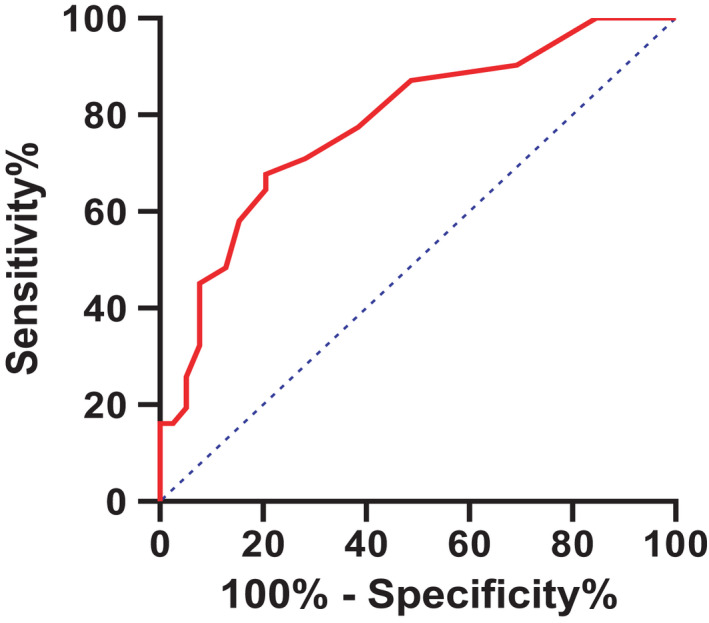
ROC curve of PCT

The ROC curve of PCT was constructed by taking the poor prognosis group as the study group and the favorable prognosis group as the control group. The analysis of ROC curve showed that the area under the ROC curve of PCT for poor prognosis of HSOS was 0.781, and the yoden index was 0.472. When the PCT value was 0.135, the diagnostic value for the poor prognosis of HSOS patients reached the maximum, and the sensitivity and specificity at this time were 67.7% and 79.5%, respectively (as shown in Figure 2).

## 4 DISCUSSION

Hepatic sinusoidal obstruction syndrome (HSOS), also known as hepatic veno‐occlusive disease (HVOD), has gradually attracted the attention of researchers.^23^, ^24^, ^25^, ^26^ The clinical manifestations of HSOS are typically the classic triad of weight gain, painful hepatomegaly, and jaundice. HSOS usually occurs in patients who have received cytoreductive therapy prior to HSCT or oxaliplatin‐containing chemotherapy for colorectal cancer.^9^, ^25^, ^26^, ^27^ In China, the primary cause of HSOS is the intake of herbals containing PA.^20^, ^28^, ^29^ It has been reported that the mortality of PAs‐induced HSOS ranges from 16% to 40%,^30^, ^31^, ^32^, ^33^ and the common cause of death is liver failure.^22^, ^24^ Previous studies aimed to find some useful indicators to predict the poor prognosis of HSOS, such as total bilirubin, aspartate aminotransferase, and blood pyrrole‐protein adducts (PPA) concentration. In this study, 70 patients were separated into two groups according to CPT scores, and WBC, NE, ALT, AST, TBIL, ALB, PT INR, D‐Dimer, and PCT were analyzed in each group. We found that PCT levels were significantly different in the two groups and speculated that it has the potential to be a prognostic indicator of HSOS.

Platelets are cytoplasmic fragments of megakaryocytes that play a role in inflammation and hemostasis. Platelet hematocrit (PCT) is the product of mean platelet volume (MPV) and platelet count (PLT), and its role in liver disease remains unclear. Many studies have found that platelet index is related to thrombotic diseases, infections, malignancies, inflammatory diseases, and trauma.^26^ At present, it has been reported that PCT can be used as a potential prognostic marker for patients with liver fibrosis and deterioration of liver failure.^34^, ^35^ Based on data analysis, we found that higher PCT levels were associated with poor prognosis in PA‐HSOS patients (*p* < 0.01). The AUC of PCT predicting poor prognosis of HSOS was 0.781, and the sensitivity and specificity were 67.7% and 79.5%, respectively, indicating the importance of PCT in assessing the prognosis of HSOS. Moreover, PCT is one of the routine blood examination items with low price and convenient detection and can also be standardized in rural hospitals.

There are still some limitations in this study. On the one hand, it was a retrospective study with a smaller sample size. On the other hand, some patients in this study were combined with infection, which may have an impact on the results.

To sum up, bilirubin level and refractory ascites are still the main indicators for evaluating the prognosis of HSOS. This study confirmed that PCT is related to the prognosis of HSOS and may have certain reference value for prognostic evaluation. For regions with relatively poor medical conditions, the price of PCT testing is relatively advantageous. Currently, HSOS still faces many problems and challenges and more large‐scale prospective studies are also needed to verify the importance of PCT in the assessment of HSOS prognosis.

## CONFLICT OF INTEREST

The authors declare that there are no conflicts of personal and funding interest.

## Data Availability

The data used to support the results of this study are available from the corresponding author upon request.

## References

[1] Friedman SL . Focus: a bad transition and a good step forward. Hepatol. 2012;57:935‐936. 10.1016/j.jhep.2012.08.015 22922569

[2] Tallman MS , McDonald GB , DeLeve LD , et al. Incidence of sinusoidal obstruction syndrome following Mylotarg (gembuzumab ozogamicin): a prospective observational study of 482 patients in routine clinical practice. Int J Hematol. 2013;97:456‐464. 23460018 10.1007/s12185-013-1275-2

[3] Yeong ML , Swinburn B , Kennedy M , Nicholson G . Hepatic veno‐occlusive disease associated with comfrey ingestion. J Gastroenterol Hepatol. 1990;5:211‐214. 2103401 10.1111/j.1440-1746.1990.tb01827.x

[4] Consolato S , Bernhard B , Otwin L , Hofmann WJ . Fatal course of veno‐occlusive disease of the liver (endophlebitis hepatica obliterans) in a preterm infant. Pathol Res Pract. 1999;195:847‐851. 10631721 10.1016/S0344-0338(99)80108-3

[5] Wang W , Yang X , Chen Y , et al. Seneciphylline, a main pyrrolizidine alkaloid in Gynura japonica, induces hepatotoxicity in mice and primary hepatocytes via activating mitochondria‐mediated apoptosis. J Appl Toxicol. 2020;40(11):1534‐1544. 32618019 10.1002/jat.4004

[6] Fan CQ , Crawford JM . Sinusoidal obstruction syndrome (hepatic veno‐occlusive disease). J Clin Exp Hepatol. 2014;4(4):332‐346. 25755580 10.1016/j.jceh.2014.10.002 PMC4298625

[7] DeLeve LD , McCuskey RS , Wang X , et al. Characterization of a reproducible rat model of hepatic veno‐occlusive disease. Hepatology. 1999;29:1779‐1791. 10347121 10.1002/hep.510290615

[8] DeLeve LD , Shulman HM , McDonald GB . Toxic injury to hepatic sinusoids: sinusoidal obstruction syndrome (veno‐occlusive disease). Semin Liver Dis. 2002;22:27‐42. 11928077 10.1055/s-2002-23204

[9] Mohty M , Malard F , Abecassis M , et al. Sinusoidal obstruction syndrome/venoocclusive disease: current situation and perspectives: a position statement from the European Society for Blood and Marrow Transplantation (EBMT). Bone Marrow Transplant. 2015;50:781‐789. 25798682 10.1038/bmt.2015.52 PMC4456788

[10] Piccin A , Sartori MT , Bisogno G , et al. New insights into sinusoidal obstruction syndrome. Intern Med J. 2017;47:1173‐1183. 28707749 10.1111/imj.13550

[11] Aktaş G , Sit M , Tekce H , et al. Mean platelet volume in nasal polyps. West Indian Med J. 2013;62(6):515‐5188. 24756737 10.7727/wimj.2013.011

[12] Bilgin S , Aktas G , Kahveci G , et al. Does mean platelet volume/lymphocyte count ratio associate with frailty in type 2 diabetes mellitus? Bratisl Lek Listy. 2021;122(2):116‐119. 33502879 10.4149/BLL_2021_017

[13] Dagistan Y , Dagistan E , Gezici AR , et al. Could red cell distribution width and mean platelet volume be a predictor for lumbar disc hernias? Ideggyogy Sz. 2016;69(11–12):411‐414. 29733559 10.18071/isz.69.0411

[14] Ustaoglu M , Aktas G , Avcioglu U , Bas B , Bahceci BK . Elevated platelet distribution width and red cell distribution width are associated with autoimmune liver diseases. Eur J Gastroenterol Hepatol. 2021;33:e905‐e908. doi:10.1097/MEG.0000000000002296 34643621

[15] Vaghari‐Tabari M , Moein S , Qujeq D , Kashifard M , Alaoddolehei H , Hajian‐Tilaki K Sensitivity and specificity of mean platelet volume as a laboratory marker for irritable bowel syndrome: can it be added to Rome criteria? Afr J Lab Med. 2020;9(1):1001. 33392049 10.4102/ajlm.v9i1.1001 PMC7756523

[16] Harifi G , Sibilia J . Pathogenic role of platelets in rheumatoid arthritis and systemic autoimmune diseases. Saudi Med J. 2016;37(4):354‐360. 27052277 10.15537/smj.2016.4.14768 PMC4852012

[17] Martin S , Mustata T , Enache O , et al. Platelet activation and inflammation in patients with papillary thyroid cancer. Diagnostics (Basel). 2021;11(11):1959. 34829306 10.3390/diagnostics11111959 PMC8624142

[18] Dignan FL , Wynn RF , Hadzic N , et al. BCSH/BSBMT guideline: diagnosis and management of veno‐occlusive disease (sinusoidal obstruction syndrome) following haematopoietic stem cell transplantation. Br J Haematol. 2013;163(4):444‐457. 24102514 10.1111/bjh.12558

[19] European Association for the Study of the Liver . EASL clinical practice guidelines: vascular diseases of the liver. J Hepatol. 2016;64(1):179‐202. 26516032 10.1016/j.jhep.2015.07.040

[20] Zhuge Y , Liu Y , Xie W , et al. Expert consensus on the clinical management of pyrrolizidine alkaloid‐induced hepatic sinusoidal obstruction syndrome. J Gastroenterol Hepatol. 2019;34:634‐642. 30669184 10.1111/jgh.14612

[21] Kan X , Ye J , Rong X , et al. Diagnostic performance of contrast‐enhanced CT in pyrrolizidine alkaloids‐induced hepatic sinusoidal obstructive syndrome. Sci Rep. 2016;6(1):37998. 27897243 10.1038/srep37998 PMC5126558

[22] Piotrowski D , Sączewska‐Piotrowska A , Jaroszewicz J , Boroń‐Kaczmarska A . Predictive power of model for end‐stage liver disease and child‐Turcotte‐Pugh score for mortality in cirrhotic patients. Clin Exp Hepatol. 2018;4(4):240‐246. 30603671 10.5114/ceh.2018.80125 PMC6311743

[23] Plessier A , Rautou PE , Valla DC . Management of hepatic vascular diseases. J Hepatol. 2012;56(Suppl 1):S25‐S38. 22300463 10.1016/S0168-8278(12)60004-X

[24] Northup PG , Garcia‐Pagan JC , Garcia‐Tsao G , et al. Vascular liver disorders, portal vein thrombosis, and procedural bleeding in patients with liver disease: 2020 practice guidance by the American association for the study of liver diseases. Hepatology. 2021;73(1):366‐413. 33219529 10.1002/hep.31646

[25] Zhu C , Ren X , Liu D , Zhang C . Oxaliplatin‐induced hepatic sinusoidal obstruction syndrome. Toxicology. 2021;460:152882. 34352347 10.1016/j.tox.2021.152882

[26] Morine Y , Shimada M , Utsunomiya T . Evaluation and management of hepatic injury induced by oxaliplatin‐based chemotherapy in patients with hepatic resection for colorectal liver metastasis. Hepatol Res. 2014;44:59‐69. 10.1111/hepr.12107 23551330

[27] Robinson SM , Mann J , Vasilaki A , et al. Pathogenesis of FOLFOX induced sinusoidal obstruction syndrome in a murine chemotherapy model. J Hepatol. 2013;59:318‐326. 23624001 10.1016/j.jhep.2013.04.014 PMC3710969

[28] Wang JY , Gao H . Tusanqi and hepatic sinusoidal obstruction syndrome. J Dig Dis. 2014;15:105‐107. 24528632 10.1111/1751-2980.12112

[29] Wang X , Qi X , Guo X . Tusanqi‐related sinusoidal obstruction syndrome in China: a systematic review of the literatures. Medicine (Baltimore). 2015;94:e942. 26061322 10.1097/MD.0000000000000942 PMC4616462

[30] Zhuge YZ , Wang Y , Zhang F , et al. Clinical characteristics and treatment of pyrrolizidine alkaloid‐related hepatic vein occlusive disease. Liver Int. 2018;38(10):1867‐1874. 29297975 10.1111/liv.13684

[31] Wang Y , Qiao D , Li Y , Xu F . Risk factors for hepatic veno‐occlusive disease caused by Gynura segetum: A retrospective study. BMC Gastroenterol. 2018;18:156. 30367628 10.1186/s12876-018-0879-7 PMC6204041

[32] Guo T , Li X , Yang X , et al. Gadoxetic acid‐enhanced hepatobiliary‐phase magnetic resonance imaging for pyrrolizidine alkaloid‐induced hepatic sinusoidal obstruction syndrome and association with liver function. Sci Rep. 2019;9:1231. 30718698 10.1038/s41598-018-37775-1 PMC6362127

[33] Gao H , Ruan JQ , Chen J , et al. Blood pyrrole‐protein adducts as a diagnostic and prognostic index in pyrrolizidine alkaloid‐hepatic sinusoidal obstruction syndrome. Drug Des Devel Ther. 2015;9:4861‐4868. 10.2147/DDDT.S87858 PMC4554430 26346783

[34] Vijayakumar S , Viswanathan S , Jain D . Utility of platelet indices in alcoholic hepatitis: a retrospective study. Porto Biomed J. 2020;5(5):e082. 33195872 10.1097/j.pbj.0000000000000082 PMC7657577

[35] Michalak A , Cichoż‐Lach H , Guz M , Kozicka J , Cybulski M , Jeleniewicz W . Plateletcrit and mean platelet volume in the evaluation of alcoholic liver cirrhosis and nonalcoholic fatty liver disease patients. Biomed Res Int. 2021;15(2021):8867985. 10.1155/2021/8867985 PMC7901043 33644233

